# Breast cancer after kidney transplantation: a single institution review

**DOI:** 10.1186/1477-7819-11-77

**Published:** 2013-03-22

**Authors:** Hee-Yong Kwak, Byung-Joo Chae, Ja-Seong Bae, Sang-Seol Jung, Byung-Joo Song

**Affiliations:** 1 Department of Surgery, Catholic University of Korea, College of Medicine, Seoul, Republic of Korea

**Keywords:** Breast cancer, Prognosis, Transplants

## Abstract

**Background:**

Improvements in immunosuppression have resulted in long life expectancy of kidney transplants. Unfortunately, the incidence of post-transplant malignancy (PTM) is increasing. The aim of this study was to evaluate the nature and stage-specific prognosis of post-transplant breast cancer (PTBC) compared with breast cancer in the general population, and to suggest optimal treatment strategies.

**Methods:**

A database of 2,139 consecutive kidney transplant patients was reviewed;11 of the patients developed breast cancer. These 11 PTBC cases underwent operations between 1999 and 2011. Next, 2,554 breast cancer patients treated in the same period were reviewed. Kaplan–Meier curves and the log-rank test were used to assess stage-specific survival of breast cancer in our hospital.

**Results:**

In total, 142 cases experienced post-transplant malignancy (PTM; 6.6%) and 11 (0.5%) developed PTBC. No one required an adjusted dose of immunosuppressive agent. Two stage III patients died. For all breast cancer patients, 5-year survival by stage was 97.7% for stage I, 92.9% for stage II, 78.6% for stage III, and 49.9% for stage IV. The 5-year survival for expected stage III-specific survival was 66.7% and no significant statistical difference was seen compared to that of the total breast cancer patients (*P* = 0.213).

**Conclusions:**

The prognosis of PTBC was comparable to that of the general population. These results suggest that the use of immunosuppressants per se does not adversely affect breast cancer.

## Background

As immunosuppressive agents have continued to improve, the lifespans of recipients after renal transplantation have been prolonged. Unfortunately, immunosuppressed renal transplant patients have a higher incidence of carcinomas than the general population [1-3].

The treatment of breast cancer in the transplant population is somewhat complicated by factors such as determining the correct dose of immunosuppressant, survival of transplant grafts, and doses of adjuvant chemotherapy. However, there is limited information regarding the treatment of this disease. Although the number of cases was small, we sought to evaluate the nature and prognosis of post-transplant breast cancer (PTBC) and to suggest optimal strategies based on our experience and a review of the literature.

## Methods

### Patient cohort

A total of 2,139 consecutive patients with chronic renal failure underwent kidney transplantation in our hospital between March 1999 and December 2011. All clinical and pathological features of the recipients of renal transplants were collected from the database of Seoul St. Mary’s Hospital. Among these patients, 142 experienced post-transplant malignancy (PTM) and 11 were diagnosed with post-transplant breast cancer (PTBC). These 11 patients underwent operations between 1999 and 2011.

In the meantime, 2,554 patients were diagnosed with invasive breast cancer and underwent operations between 1999 and 2011. Exclusion criteria were patients with no pathological TNM status due to mass excision at an outside hospital, in situ breast cancer, previous breast cancer surgery, prior radiation, distant metastasis, and prior axillary surgery. Thus, in total, 1,722 patients were reviewed. All protocols were approved by the institutional review committee of Seoul St. Mary’s Hospital, Catholic University of Korea (KC12RISI0626) and met the guidelines of the responsible governmental agency.

### Statistical analyses

The primary endpoint was death (overall survival). All patients who underwent an operation according to the standard protocols used at our hospital were treated with adjuvant chemotherapy and/or radiotherapy. Overall survival (OS) was defined as the time from the diagnosis of breast cancer to death. Statistical significance was determined using the chi-squared test. Survival rates were calculated by the Kaplan–Meier method and compared with the log-rank test, and then by stage of disease. *P* values < 0.05 were deemed to indicate statistical significance.

## Results

Overall, 142 subjects experienced post-transplant malignancy (PTM; 6.6%). All cases were confirmed both clinically and histologically. Of the 142 PTM patients, 11 (0.5%) developed PTBC. These 11 patients underwent operations between 1999 and 2011. The mean ages of the PTM and PTBC cases were 54.7 ± 20.7 and 35 ± 8.0 years, respectively. The mean times before the occurrence of cancer were 110.7 ± 71.3 and 136.5 ± 101.2 months, respectively (Table 1).

**Table 1:** Comparison of demographics

	**KT**	**PTM**	**PTBC**
*n*	2,139	142	11
Age, years (mean ± SD)	51.0 ± 17.1	54.7 ± 20.7	35 ± 8.0
Male: female ratio	65.0 : 35.0	54.9 : 45.1	9.1 : 90.9
Mean time of occurrence, months (mean ± SD)		110.7 ± 71.3	136.5 ± 101.2

KT: kidney transplantation; PTBC: post-transplant breast cancer; PTM: post-transplant malignancy.

Of the 11 PTBC patients, 9 patients take 200 mgNeoral capsule(cyclosporine capsules) per day. One patient takes azathioprine (patient number 9) and one other patient takes FK506 (tacrolimus) (patient number 1).

Unlike other studies [4-7], gastrointestinal cancers, such as stomach and colon cancer, developed in 39 of the 142 PTM patients (27.5%) and were the most frequent. Other cancers were lymphomas (14.8%), urogenital cancers (12%), gynecological cancers (8.5%), and breast cancer (7.7%).

Table 2 shows the clinicopathological features of the 11 PTBC patients. The median age at diagnosis of breast cancer was 49 years. Five patients underwent modified radical mastectomies, four had simple mastectomies with sentinel lymph node biopsies, one had a wide excision with sentinel lymph node biopsy, and one underwent an excision biopsy only. Five patients received adjuvant chemotherapy and one, neoadjuvant chemotherapy. The stages were I for five, II for one, III for four, and unknown for one patient. Five underwent adjuvant endocrine therapy and two, radiation therapy. One experienced post-operative bleeding. After 30 months median follow-up, two have died.

**Table 2:** Clinicopathological features of the 11 PTBC patients

**Patient**	**Age**	**Duration to diagnosis** (**months**)	**Procedure**	**Stage**	**Positivity of hormone receptor**	**Type of chemotherapy**^**a**^	**Recurrence**	**Status**	**Follow**-**up duration** (**months**)
1	42	42	SM with SLNB	I	+	-	-	Alive	127
2	49	6	MRM	I	+	-	-	Alive	119
3	28	127	MRM	III	-	CAF(×6)	Bone, lung	Dead	95
4	24	134	Excision biopsy	Unknown	-	-	-	Alive	146
5	40	177	SM with SLNB	I	-	-	-	Alive	101
6	43	181	MRM	III	-	CMF(×6) + AT(×4)	Bone, lung, liver	Dead	30
7	35	89	MRM	III	-	AC(×4) + T(×4)	-	Alive	49
8	28	354	SM with SLNB	II	+	AC(×4)	-	Alive	8
9	29	176	WE with SLNB	I	+	-	-	Alive	37
10	29	208	SM with SLNB	I	-	-	-	Alive	28
11	38	8	MRM	III	+	AC(×4)	-	Alive	17

^a^C: cyclophosphamide, M: methotrexate, F: 5-fluorouracil, A: adriamycin, T: taxotere.

MRM: modified radical mastectomy; SLNB: sentinel lymph node biopsy;SM: simple mastectomy;WE: wide excision.

**Figure 1:**
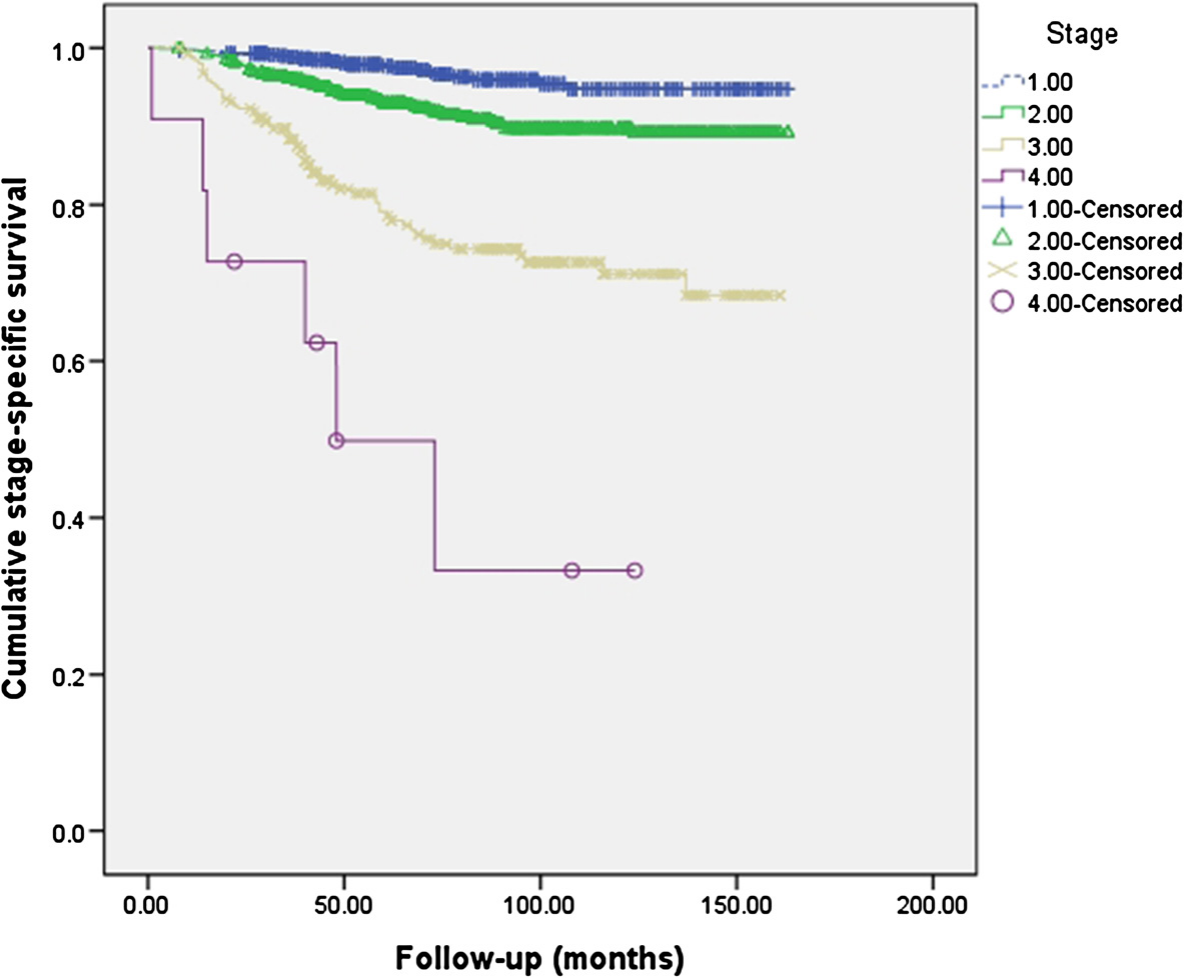
**Cumulative stage**-**specific survival of general population for breast cancer patients.** Five-year survival for each stage was 97.7% for stage I, 92.9% for stage II, 78.6% for stage III, and 49.9% for stage IV.

Figure 1 shows stage-specific survival of 1,722 breast cancer patients who underwent breast surgery between 1999 and 2011 at our hospital. Five-year overall survival was 92.6%. Five-year survival for each stage was 97.7% for stage I, 92.9% for stage II, 78.6% for stage III, and 49.9% for stage IV. In our study, the median follow-up period for PTBC patients was 49 months (range: 8–146 months) and two of the 11 PTBC patients died, after 30 and 95 months of follow-up. Cumulative stage-specific survival for the general population of breast cancer patients was compared with the PTBC patients (Table 3). Although based on a small number of cases, the 5-year survival for expected stage III-specific survival was 66.7% and no significant statistical difference was seen compared to that of the general population of breast cancer patients (*P* = 0.213).

**Table 3:** **Comparison of cumulative stage**-**specific survival**

	**PTBC**^**a**^	**General population of breast cancer**^**a**^	***P*value**
Stage I	101.0 (28–127)	71.0 (6–163)	0.690
Stage II	8.0 (8)	77.0 (3–163)	0.766
Stage III	39.5 (17–95)	70.0 (8–161)	0.213

^a^Data are median months of survival with range in parentheses.

PTBC:post-transplant breast cancer.

## Discussion

Patients undergoing kidney transplantation have a higher risk of developing cancer as a result of immunosuppressive treatment [1,3,6]. Thus, there is the possibility that the population of breast cancer sufferers after kidney transplantation will increase. This is the first report to describe the characteristics of PTBC and to compare the incidence of breast cancer in PTBC patients with that in the general population.

Few reports have focused on breast cancer in the kidney transplant population. Vachtova et al. [8] reported PTBC without a reduction in the dose of immunosuppressive agent. However, this was a case report and one experience does not explain all patients. Popov et al. [5] and Veroux et al. [9] also reported one PTBC patient; however, they did not focus on the nature of the breast cancer and no description of the disease was provided. Thus, no comparison with the general population was made.

For treatingPTM, adjustment of the immunosuppressant agent(s) must be considered. The management of immunosuppression is also an area of concern because few data are available to guide decisions. When surgery is needed, dialysis access should be considered [10]. Renal graft function should be considered before chemotherapy. Among chemoagents, paclitaxel is an important treatment option in patients with impaired renal graft function [11]. There are few data regarding radiation or hormonal therapy.

A large cohort-based study reported that 5-year net survival was 84% in the US and 81% in Europe [12], but this was age-standardized net survival. Women had a 5-year overall survival (OS) of 94% for stage 0, 90% for stage I, 82% for stage II, 56.9% for stage III, and 19% for stage IV [13]. In our study, median follow-up was 68 months, and two of the 11 PTBC patients died, after 30 and 95 months, of follow-up. For stage III patients, the probability of surviving was approximately 66.7% at 5 years after diagnosis, comparable to that reported by Greif et al. [13].

The younger age of PTBC patients in our study is probably due to the small number of patients. When more patients are evaluated, the age of PTBC patients might increase. This is because the majority of breast cancer cases occur in women older than 50, with the highest incidence rate occurring between the ages of 70 and 75 years [14].

Agraharkar et al. [15] found that the relative risk of breast cancer in a post-transplant population was approximately 0.7 compared with that of the general population. This is consistent with our findings. Also, Buell et al. [16] demonstrated that immunosuppression may not increase the incidence, but may increase the biological aggressiveness of breast cancer. However, no adjustment of immunosuppression was made in our 11 patients and overall survival was comparable to that of general breast cancer patients.

The management of immunosuppression is an area of concern when a cancer is diagnosed. The overall amount of immunosuppression is a factor that may lead to an increased incidence of malignancies after renal transplantation [17]. Decreased or cessation of immunosuppression has been associated with remission of lymphoproliferative processes. However, data forthe therapeutic benefit of reducing immunosuppression in patients with solid tumors is anecdotal [10]. Moreover, in a previous report by Marcen et al. [18], cyclosporine therapy was not a risk factor for any type of malignancy.

This study has some limitations. First, the small numbers cannot provide a proxy for the whole PTBC population. Second, the median follow-up for PTBC was short. This was due to the fact that most PTM is reported late (median, 134 months) after transplantation. Finally, this was a retrospective study and several confounding factors were not considered.

However, this study is useful because it is a rare study on the prognosis of breast cancer patients after kidney transplantation. To our knowledge, there has been no previous research on the prognosis of PTBC patients. Also, we recommend that modifying dose of immunosuppressant after transplantation may not be necessary with regard to the breast cancer.

## Conclusion

In conclusion, PTBC patients did not exhibit a poorer prognosis than the general breast cancer population. Transplant physicians should screen post-transplant patients to identify cancers at an early stage. With regard to PTBC, the best approach is a combination of breast surgery, transplant surgery, and nephrology.

## Abbreviations

MRM: modified radical mastectomy; OS: overall survival; KT: kidney transplantation; PTBC: post-transplant breast cancer; PTM: post-transplant malignancy; SLNB: sentinel lymph node biopsy; SM: simple mastectomy; WE: wide excision.

## Competing interests

The authors have declared no conflicts of interest.

## Authors’ contributions

HY Kwak contributed mainly in the design, literature review, and writing of the article. The data were collected and assembled by HY Kwak and BJ Chae. BJ Chae, JS Bae, and SS Jung gave valuable advice and edited the discussion. Both SS Jung and BJ Song provided administrative support. BJ Song has given final approval for the version to be published. All authors read and approved the final manuscript.

## References

[B1] Catena F Nardo B Livianod’Arcangelo G Stefoni S Arpesella G Faenza A Cavallari A *De novo* malignancies after organ transplantation Transplant Proc 2001 33 1858 1859 10.1016/S0041-1345(00)02724-X 11267542

[B2] Winkelhorst JT Brokelman WJ Tiggeler RG Wobbes T Incidence and clinical course of *de*-*novo* malignancies in renal allograft recipients Eur J SurgOncol 2001 27 409 413 10.1053/ejso.2001.1119 11417989

[B3] London NJ Farmery SM Will EJ Davison AM Lodge JP Risk of neoplasia in renal transplant patients Lancet 1995 346 403 406 10.1016/S0140-6736(95)92780-8 7623570

[B4] Marcen R Galeano C Fernandez-Rodriguez A Jimenez-Alvaro S Teruel JL Rivera M Burgos FJ Quereda C Effects of the new immunosuppressive agents on the occurrence of malignancies after renal transplantation Transplant Proc 2010 42 3055 3057 10.1016/j.transproceed.2010.08.002 20970609

[B5] Popov Z Ivanovski O Kolevski P Stankov O Petrovski D Cakalaroski K Ivanovski N *De novo* malignancies after renal transplantation–a single-center experience in the Balkans Transplant Proc 2007 39 2589 2591 10.1016/j.transproceed.2007.08.022 17954184

[B6] Pedotti P Cardillo M Rossini G Arcuri V Boschiero L Caldara R Cannella G Dissegna D Gotti E Marchini F Incidence of cancer after kidney transplant: results from the North Italy transplant program Transplantation 2003 76 1448 1451 10.1097/01.TP.0000083897.44391.E8 14657684

[B7] Stratta P Morellini V Musetti C Turello E Palmieri D Lazzarich E Cena T Magnani C Malignancy after kidney transplantation: results of 400 patients from a single center Clin Transplant 2008 22 424 427 10.1111/j.1399-0012.2008.00802.x 18312442

[B8] Vachtova M Treska V Suvova B Hes O Ebelenderova D Breast carcinoma in a female patient after kidney transplantation–a case review RozhlChir 2009 88 687 690 20662452

[B9] Veroux M Puliatti C Fiamingo P Cappello D Macarone M Puliatti D Vizcarra D Gagliano M Veroux P Early *de novo* malignancies after kidney transplantation Transplant Proc 2004 36 718 720 10.1016/j.transproceed.2004.03.021 15110643

[B10] Self M Dunn E Cox J Brinker K Managing breast cancer in the renal transplant patient: a unique dilemma Am Surg 2006 72 150 153 16536246

[B11] Luftner D Flath B Akrivakis C Prinz B Mergenthaler HG Wernecke KD Possinger K Feasibility of dose-intensified paclitaxel after chemotherapy-induced renal insufficiency in a patient with renal transplantation Eur J Cancer 1999 35 325 10448279 10.1016/s0959-8049(98)00265-2

[B12] Allemani C Sant M Weir HK Richardson LC Baili P Storm H Siesling S Torrella-Ramos A Voogd AC Aareleid T Breast cancer survival in the US and Europe: a CONCORD high-resolution study Int J Cancer 2012 132 1170 1181 22815141 10.1002/ijc.27725 PMC4706735

[B13] Greif JM Pezzi CM Klimberg VS Bailey L Zuraek M **Gender differences in breast cancer: analysis of 13,000 breast cancers in men from the national cancer data base** Ann SurgOncol 2012 19 3199 3204 10.1245/s10434-012-2479-z 22766989

[B14] Ries LAG Melbert D Krapcho M Stinchcomb DG Howlader N Horner MJ Mariotto A Miller BA Feuer EJ Altekruse SF Lewis DR Clegg L Eisner MP Reichman M Edwards BK SEER Cancer Statistics Review 2008 Breast. Based on November 2007 SEER data submission, posted to the SEER website, 2008. National Cancer Institute. Besthesda, MD. Retrieved March 13, 2009: Edwards, B.K http://seer.cancer.gov/csr/1975_2005/

[B15] Agraharkar ML Cinclair RD Kuo YF Daller JA Shahinian VB Risk of malignancy with long-term immunosuppression in renal transplant recipients Kidney Int 2004 66 383 389 10.1111/j.1523-1755.2004.00741.x 15200447

[B16] Buell JF Hanaway MJ Trofe J Gross TG Beebe TM Alloway RR First MR Woodle ES *De novo* breast cancer in renal transplant recipients Transplant Proc 2002 34 1778 1779 10.1016/S0041-1345(02)03063-4 12176572

[B17] Sells RA Iatrogenic problems caused by immunosuppressive drugs in transplant recipients Transplant Proc 1998 30 14 10.1016/S0041-1345(98)01534-6

[B18] Marcen R Pascual J Tato AM Teruel JL Villafruela JJ Fernandez M Tenorio M Burgos FJ Ortuno J Influence of immunosuppression on the prevalence of cancer after kidney transplantation Transplant Proc 2003 35 1714 1716 10.1016/S0041-1345(03)00669-9 12962768

